# Varicella in Poland: economic burden in children 1–12 years of age in Poland, 2010–2015

**DOI:** 10.1186/s12889-018-5298-8

**Published:** 2018-03-27

**Authors:** Jacek Wysocki, Ilona Malecka, Joanna Stryczynska-Kazubska, Emmanouil Rampakakis, Barbara Kuter, Lara J. Wolfson

**Affiliations:** 1 Poznan University of Medical Sciences, Collegium Maius, Fredry 10, 61-701 Poznań, Poland; 2 JSS Medical Research, 9400 Henri-Bourassa O, Montreal, QC H4S 1N8 Canada; 3 Merck & Co., Inc., 2000 Galloping Hill Rd, Kenilworth, NJ 07033 USA

**Keywords:** Varicella, Burden of illness, Vaccination, Retrospective studies, Pediatric, Varicella vaccine

## Abstract

**Background:**

The safety and efficacy of live-attenuated varicella zoster virus (VZV) vaccines in preventing varicella and reducing associated morbidity and mortality in real-world have been previously shown. In Poland, VZV vaccination is only mandatory for certain high-risk individuals. Here, we have conducted an evaluation of the clinical and economic burden of varicella in Poland.

**Methods:**

Multicenter, retrospective chart review of varicella inpatients and outpatients aged 1–12 years with a primary diagnosis between 2010 and 2015. Varicella-related outcomes included the incidence of complications, the proportion of patients reporting healthcare resource utilization (HCRU), and frequency of HCRU. Direct costs were derived from per patient resource use multiplied by unit costs, and indirect costs were calculated as loss of revenue of caregivers reporting work days missed. The overall annual cost of varicella in Poland was estimated based on the calculated direct and indirect costs per case and the estimated number of varicella cases. All costs are presented in 2015 Polish złoty (PLN) / Euros (€).

**Results:**

A total of 150 children with varicella were included, of which 75 were outpatients and 75 were inpatients with a mean (± SD) age of 3.9 (±2.6) and 4.2 (±2.3) years, respectively. Complications were experienced by 14.7% of outpatients and 82.7% of inpatients, of which the most common were skin and soft tissue infections and dehydration. The rate of HCRU was as follows: over-the-counter medications (80.0% outpatients, 81.3% inpatients), prescription medications (80.0% outpatients, 93.3% inpatients), tests/procedures (0.0% outpatients, 69.3% inpatients), and allied health professional consults (0.0% outpatients, 24.0% inpatients). Total (direct and indirect) cost per varicella case was 5013.3 PLN (€ 1198.1) for inpatients and 1027.2 PLN (€ 245.5) for outpatients, resulting in an estimated overall annual (2015) cost of varicella in Poland of 178,198,320 PLN (€ 42,588,385) among children aged 1–15 years.

**Conclusions:**

Significant clinical and economic burden is associated with varicella in Poland. These results may be used to foster discussion related to the implications of implementing routine VZV vaccination in Poland.

## Background

Varicella, or chickenpox, is a highly infectious disease caused by the varicella-zoster virus (VZV) that predominantly occurs in childhood. Symptoms that follow varicella infection usually include fever, malaise, headache, and abdominal pain, which typically appear following a 10- to 21-day incubation period [1, 2]. The majority of cases are associated with a generalized pruritic vesicular rash, however infection can occasionally result in complications, some of the most common being neurologic, skin and soft tissue, gastrointestinal or lower respiratory involvement, and pneumonia [3–5].

Approximately 2 to 16 cases of varicella are reported for every 1000 persons worldwide [6–8], with regional variations owing to differences in age, immunosuppression, and climate. Prior to universal vaccination, varicella seroprevalence rates across European countries have been found to exceed 90% by the age of 15, with the exception of Greece (86.6%) and Italy (85.3%) [9]. In Poland, recent estimates of varicella incidence report 575.90 cases per 100,000 persons in 2014 and 487.26 cases per 100,000 persons in 2015 (preliminary data), for a total of 221,628 and 187,518 cases, respectively [10, 11]. Due to the fact that patients without significant complications may opt out of medical consultation, the reported number of cases is likely underestimated. Studies performed in Poland have also demonstrated that the overall seroprevalence among the populace aged 1–19 years climbs steadily from 26% in 1 year olds, up to 82% by the age of 10, peaking at 98% in 19 year old young adults [12]. This implies that the annual number of cases could be similar to the size of the Polish birth cohort (365,000 in 2015) in the absence of an immunization program [13].

In Poland, treatment guidelines for immunocompetent patients presenting with varicella state that these patients should be treated within the framework of primary healthcare, including symptomatic treatment with antipyretics, antipruritic, and analgesic drugs, and gentle drying of the skin after bathing [14]. For patients at risk of or experiencing complications, acyclovir (ACV) antiviral treatment is recommended.

Varicella vaccines, first licensed in the 1980s, usually contain a live-attenuated virus of the Oka strain, [15–20] which are well tolerated and effective in routine practice [21–26]; cost-effectiveness has also been evaluated and confirmed in pediatric populations [27, 28]. These vaccines are available in several countries for use in children typically over the age of 12 months who present without any contraindications. Several countries have witnessed significant declines in varicella associated morbidity after including the vaccine as part of their immunization programs. For example, the US, Canada, Uruguay, Spain, Germany, Italy, and Australia have observed declines of > 99.2%, 93.0%, 94.0%, 95.2%, 77.6%, 84.0%, and 76.8%, respectively, in hospitalizations associated with varicella after introducing the vaccine in their national vaccination program [29]. A study conducted in Italy between 2001 and 2010 also demonstrated decreasing varicella incidence and associated hospitalizations with increasing vaccine coverage rates in the first 3 Italian regions to implement universal varicella vaccination [30].

In Poland, all children under the age of 18 are covered by the public health care program; this includes total costs of medical care, as well prescription medications, for which the cost of medication paid for by the patient is dependent on the status of the drug within the Polish drug benefit plan. Varicella vaccines, were first granted licensure in Poland in 1999, and the vaccine is mandatory and reimbursed for children up to 12 years of age who meet specific conditions, i.e. if they are immunocompromised, in remission of acute lymphoblastic leukemia, or human immunodeficiency virus (HIV) infected, and the vaccine must be administered prior to immunosuppressive therapy or chemotherapy. It is also reimbursed for children (≤12 years) who are living in close quarters to the previously mentioned immunocompromised individuals and for children (≤12 years) who are at risk due to densely populated living conditions such as long-term care, nurseries, and orphanages. Varicella is, nonetheless, one of the recommended vaccines in the national schedule, with specific recommendations for those who have not yet had chickenpox or have not yet been vaccinated, in addition to women trying to conceive; however, it is not reimbursed as part of the country’s national immunization programme [12, 31], perhaps partly due to the limited data on the disease burden associated with varicella in Poland, particularly in the pediatric population.

The main objective of this study was to describe the burden of illness associated with varicella among children aged 1–12 years in Poland by assessing morbidity, healthcare resource utilization (HCRU), and the associated cost among those with a varicella diagnosis who had consultations as either outpatients or inpatients between 2010 and 2015. The results of this study aim to provide local evidence regarding the HCRU and costs associated with varicella with the intent to aid policy makers in Poland when assessing interventions and benefits of implementing a national varicella vaccination plan.

## Methods

### Study design

This was a multicenter, observational study that assessed the varicella-associated burden of illness through the retrospective review of patient charts and was conducted according to the generally accepted standards of Good Pharmacoepidemiology Practice (GPP). In line with the local regulations, notification to the central Ethics Committee (EC) was done for all participating sites. Patient consent was not required, as data were collected retrospectively and provided by the treating physicians in an anonymous manner, identified only by an encrypted patient number.

### Case selection

Based on the recommendations of the principal investigator, 8 potential physician sites were selected to participate in the study, of which 7 (4 hospitals and 3 private practices) agreed to participate in the study and contributed patient charts, while one site decided not to participate for unknown reasons despite preliminary interest. All 7 sites were located in urban areas [Warszawa (*n* = 3), Konin, Poznan, Bydgoszcz, Lodz]. For case selection, investigators were asked to identify patient charts in their practices, starting from the most recent year and looking back as much as 5 years, for eligible patients to include in the study. In all sites, this was achieved by use of electronic patient records, whereby patients with either “varicella”, or a combination of “varicella” and a related complication, entered in the diagnosis field, were selected for. The date of first primary varicella infection was defined as the index date, and each patient’s chart was reviewed from this date until the resolution of the disease occurred or the last date of contact, if the resolution date was unavailable.

### Study population

A total of 150 patients with a primary varicella diagnosis between March 2010 and April 2015, and aged 1-12 years at the time of diagnosis, in roughly equal numbers of outpatients and inpatients, were targeted for inclusion. In Polish routine care, varicella cases seen in an outpatient setting are typically managed by a general physician (GP) or a pediatrician, with a small portion of patients who go directly to the emergency department (ER). Although less common, patients may also be managed upon consultation at a hospital outpatient clinic. Outpatients were therefore defined as those patients who visited either the doctor’s office (family doctor, GP, pediatrician, and infectious disease specialist), or who consulted a physician during an ER (without hospitalization), or outpatient clinic visit, for varicella. Inpatients were defined as those admitted to a hospital for their primary varicella. Patients who had a second case of varicella, those with varicella as a secondary diagnosis, or those who were previously vaccinated for varicella and had a diagnosis of breakthrough varicella, were excluded from the study.

### Outcome measures

The following clinical complications due to varicella were evaluated: skin and soft tissue infection, meningitis, encephalitis, pneumonia, sepsis, acute osteomyelitis, septic arthritis, cerebellitis, keratoconjunctivitis, hepatitis, nephritis, febrile seizure, dehydration, severe pain, and coagulation disorder. Additional types of complications could also have been reported which were coded using the Medical Dictionary for Regulatory Activities (MedDRA), version 18.0, and were reported accordingly.

The following types of varicella and varicella-related HCRU were evaluated: outpatient visits, allied healthcare contacts, doctor’s visits, tests/procedures performed, prescription medications prescribed, over-the-counter (OTC) medications, hospitalizations, ER visits/stays, and intensive care unit (ICU) stays. For each HCRU parameter, the proportion of patients using each resource as well as the frequency of use were calculated; for inpatients only, the duration of hospital and/or ICU stay were estimated. The direct cost of HCRU was derived by multiplying the per patient resource utilization rate with the unit cost of each resource (ingredients-based approach), which were based on the payments of the National Health Fund to the Regional Hospital Centre for Mother and Child in Poznań, Poland. For hospital outpatient clinic costs, estimates were based on an average salary of 9000 PLN/month for a specialized physician in the public system and an average of 25 patients/day. The indirect cost was defined as the revenue loss for caregivers, which was estimated using the national average income statistics reported by the Organization for Economic Co-Operation (OECD) [32] and the number of work days missed, based on the days that an outpatient was ordered by the doctor to be absent from school and the days spent in the hospital/ICU for inpatients.

The overall cost of pediatric varicella in 2015 was calculated based on the number of varicella cases reported to the Polish Department of Prevention and Control of Infectious Diseases for 2015 (*n* = 187,518) [10], taking into account the proportion of these that were pediatric cases reported to the National Institute of Public Health, Department of Epidemiology, in 2014 (91.79%; *n* = 172, 117 pediatric cases) [11]. Number of inpatients versus outpatients was estimated from the number of hospitalizations due to varicella reported in 2014 (*n* = 1467) [11] considering the distribution of varicella across age groups, as reported to the European infectious disease surveillance system in 2010 [33], for a final pediatric hospitalization rate of 0.20%. The respective per patient direct and indirect costs, calculated as described above, were then multiplied by the estimated total number of Polish pediatric inpatients vs. outpatient cases. All costs are presented in 2015 PLN / Euros (€) [34].

### Statistical methods

All enrolled patients were included in the statistical analysis, and subgroup analysis was performed for outpatients and inpatients. Descriptive statistics were produced to address all study objectives, which included measures of central tendency (mean) and dispersion statistics (standard deviation, [SD] and 95% confidence interval [CI]) for continuous variables, and frequency distributions (number and percentage) for categorical variables. Due to the low number of cases in some outcome measures, logarithmic transformation was used for the calculation of 95% CIs. All statistical analyses were performed using SAS® software version 9.4 (SAS Institute Inc., Cary, NC, USA).

## Results

Table 1 summarizes the patient and disease characteristics of the study cohort at varicella diagnosis. A total of 75 (50.0%) outpatients and 75 (50.0%) inpatients were included in the study. The mean (±SD) outpatient age was 3.9 (±2.6) years, and for inpatients it was 4.2 (±2.3) years, with slightly more male patients enrolled in the inpatient group (54.7% and 61.3%, respectively). The distribution of patients who had < 50, 50–249, or 250–500 skin lesions during their rash outbreak was similar across both groups, the predominant category being 50 to 249 skin lesions (48.0% and 49.3%, respectively). Of note, more than 500 skin lesions were only reported for 10.7% of inpatients as opposed to no outpatients No patient in the study was considered immunocompromised.

**Table 1:** Patient and disease characteristics at varicella diagnosis

	Outpatients	Inpatients
	(*N* = 75)	(*N* = 75)
Patient Characteristics		
Age, years, mean (±SD)	3.9 (± 2.6)	4.2 (± 2.3)
Gender, n (%)		
Male	41 (54.7%)	46 (61.3%)
Female	34 (45.3%)	29 (38.7%)
Area of residence, n (%)		
Urban	64 (85.3%)	51 (68.0%)
Rural	7 (9.3%)	23 (30.7%)
BMI, kg/m^2^, mean (±SD)^a^	17.5 (**±**1.6)	16.4 (**±**2.1)
Calendar year of diagnosis, n (%)		
2010	2 (2.7%)	0 (0.0%)
2011	6 (8.0%)	0 (0.0%)
2012	8 (10.7%)	0 (0.0%)
2013	7 (9.3%)	12 (16.0%)
2014	42 (56.0%)	60 (80.0%)
2015	10 (13.3%)	3 (4.0%)
Disease Characteristics		
Maximum number of skin lesions during rash^c^, n (%)		
< 50	25 (33.3%)	18 (24.0%)
50–249	36 (48.0%)	37 (49.3%)
250–500	14 (18.7%)	12 (16.0%)
> 500	0 (0.0%)	8 (10.7%)
Patients who were immunocompromised^b^, n (%)	0 (0.0%)	0 (0.0%)

^a^Calculations based on data from 31 (41.3%) outpatients and 44 (58.7%) inpatients

^b^Patients were considered immunocompromised if they had at least one of the following conditions: HIV/AIDS, congenital immunodeficiency, received system steroids, or had any other immunocompromised condition listed in their medical history

^c^Number of varicella lesions were either extracted directly from the patient chart, or approximated from physician categorization of the severity of the skin eruption at presentation (low, moderate, severe)

**Fig. 1:**
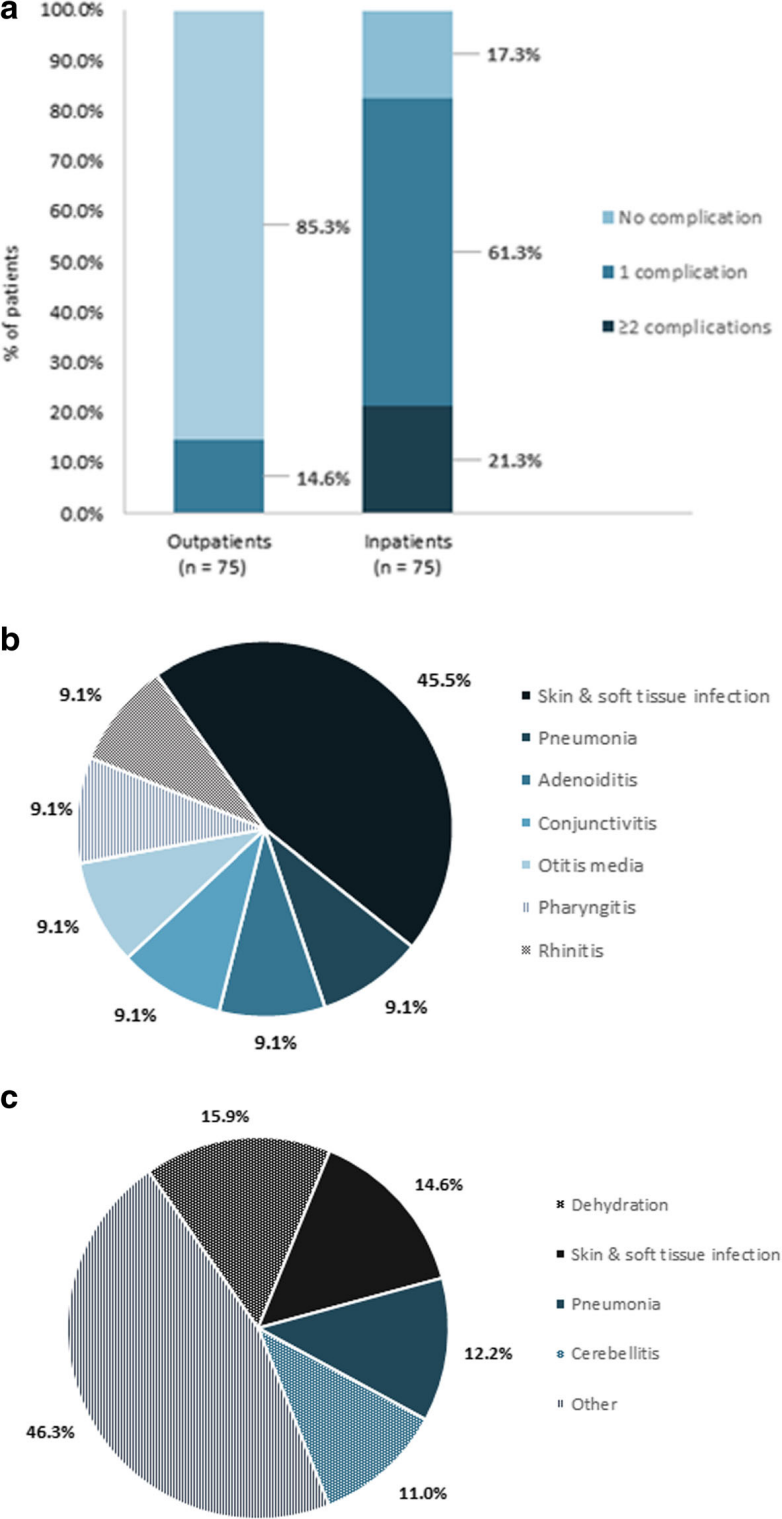
**a** Types of complications associated with varicella - Percentage of patients with complications*^§^. **b** Types of complications associated with varicella - Types of complications - Outpatients^†^. **c** Types of complications associated with varicella - Types of complications - Inpatients^†‡^. * Proportions based on the total number of patients. § Of those experiencing complications, 100% of outpatients had exactly one (*n* = 75), whereas 25.8% of inpatients experienced more than one complication (*n* = 16). † Proportions based on the total number of complications. ‡ Inpatients; other includes: sepsis, febrile seizure, and vomiting (3.7%, each); meningitis, coagulation disorder diarrhoea, otitis media, pharyngitis, scarlet fever, and upper respiratory tract infection (2.4%, each); encephalitis, acute osteomyelitis, keratoconjunctivitis, severe pain, anaemia, bronchitis, conjunctivitis, allergic dermatitis, gastritis, loss of consciousness, seizure, syncope, tonsillitis, torticollis, and urticaria (1.2%, each)

Figure 1 presents the varicella-related complications that were reported for outpatients and inpatients. Overall, the majority of inpatients (82.6%: 61.3% 1 complication and 21.3% ≥ 2 complications) experienced at least one varicella-related complication compared to 14.6% of outpatients (Fig. 1a). Of those experiencing complications, 100% of outpatients had exactly one, whereas 25.8% of inpatients experienced more than one complication. The most common complications for outpatients (Fig. 1b) were skin and soft tissue infection (45.5% of all complications), pneumonia (9.1%), adenoiditis (9.1%), conjunctivitis (9.1%), otitis media (9.1%), pharyngitis (9.1%), and rhinitis (9.1%), whereas for inpatients (Fig. 1c), frequent complications included dehydration (15.9%), skin and soft tissue infection (14.6%), pneumonia (12.2%), and cerebellitis (11.0%). The inpatient group also experienced additional complications, such as: sepsis, febrile seizure, and vomiting (3.7% of all complications, each); meningitis, coagulation disorder diarrhoea, otitis media, pharyngitis, scarlet fever, and upper respiratory tract infection (2.4% of all complications, each); encephalitis, acute osteomyelitis, keratoconjunctivitis, severe pain, anaemia, bronchitis, conjunctivitis, allergic dermatitis, gastritis, loss of consciousness, seizure, syncope, tonsillitis, torticollis, and urticaria (1.2% of all complications, each).

Table 2 reports the varicella-associated HCRU for each patient group, inclusive of the health care contact during which the varicella diagnosis was made. Among outpatients, doctor’s offices were more frequently visited (92.0% of patients; 22.7% visited more than once) compared to ERs (12.0%; 1.3% visited more than once) or hospital outpatient clinics (17.3%; 0.0% visited more than once), nevertheless the average number of times that patients used these resources was similar [1.3 (95% CI: 1.0, 1.6), 1.1 (95% CI: 0.6, 1.9), and 1.0 among users, respectively]. Following doctor’s office visits, medication was the resource used the most among outpatients, for a total of 80.0% of patients each using prescription and OTC medications, with an average use per patient of 1.2 (95% CI: 0.9, 1.4) and 1.7 (95% CI: 1.3, 2.0), respectively. Tests/procedures and allied health professional consultations were not used by any outpatient.

**Table 2:** Varicella associated healthcare resource utilization (HCRU)

Type of HCRU	Outpatients (*N* = 75)		Inpatients (*N* = 75)	
Parameter	% Patients	Mean (95% CI)^a^	% Patients	Mean (95% CI)^a^
Visits to doctor’s office	92.0%	1.3 (1.0, 1.6)	57.3%	1.2 (0.9, 1.5)
Visits to ER	12.0%	1.1 (0.6, 1.9)	5.3%	1.0 (N/C)
Visits to hospital outpatient clinic	17.3%	1.0 (N/C)	0.0%	N/A
Total outpatient visits^b^	100%	1.2 (1.1, 1.3)	60.0%	1.0 (1.0, 1.1)
Hospitalization	N/A	N/A	100%	4.7 (4.1, 5.3)
ICU stay	N/A	N/A	0.0%	N/A
Prescription medications	80.0%	1.2 (0.9, 1.4)	93.3%	2.1 (1.8, 2.5)
OTC medications	80.0%	1.7 (1.3, 2.0)	81.3%	1.8 (1.5, 2.1)
Tests/procedures	0.0%	N/A	69.3%	1.8 (1.5, 2.2)
Allied health professional consultations	0.0%	N/A	24.0%	1.1 (0.7, 1.6)

*N/A* not applicable, *N/C* not calculable

^a^Denotes the average number of times each healthcare resource was used among users; for hospitalization and hospital ICU stay, it denotes the duration of days

^b^Sum of visits to doctor’s office, ER, and hospital outpatient clinic

Among the inpatient group, the mean number of days that patients spent in the hospital was 4.7 (95% CI: 4.1, 5.3); none required admission to an ICU. The resource used most often by inpatients was medication, specifically prescription [93.3% of patients; mean number per patient: 2.1 (95% CI: 1.8, 2.5)] and OTC [81.3%; 1.8 (95% CI: 1.5, 2.1)]. Two outpatient services were used by inpatients, doctor’s office visits (57.3% of patients; 9.3% visited more than once) and ER visits (5.3%; 0.0% visited more than once), with mean number of visits per patient of 1.2 (95% CI: 0.9, 1.5) and 1.0, respectively. Tests/procedures and allied health professional consultations were used among 69.3% [mean number per patient: 1.8 (95% CI: 1.5, 2.2] and 24.0% [1.1 (95% CI: 0.7, 1.6] of inpatients, respectively.

Table 3 provides the per unit cost of the key healthcare resource parameters; Table 4 summarizes the per case direct and indirect associated costs per varicella case for outpatients and inpatients by type of resource utilized as a result of varicella. The overall mean direct cost per patient for outpatients in this study was 228.8 (95% CI: 214.1, 243.4) PLN and for inpatients was 4013.5 (95% CI: 3953.1, 4073.9) PLN. For outpatients, visits to doctor’s offices accounted for most of the overall direct cost [mean (95% CI): 200.2 (186.5, 213.9) PLN], whereas hospitalization cost [mean (95% CI): 3671.0 PLN] made up the predominant portion of the overall direct costs for inpatients. Visits to doctor’s offices were associated with the second highest cost [mean (95% CI): 124.8 (99.8, 149.7) PLN] for inpatients. The indirect cost of varicella was a considerable amount for both outpatients and inpatients, with mean costs of 798.5 (630.4, 966.5) PLN and 999.8 (849.3, 1150.2) PLN per case, respectively.

**Table 3:** Key unit costs (PLN / €) for healthcare resources

	Mean Cost
Healthcare resource	
Visits to doctor’s office^a^	PLN 217.6
	€ 52
Visits to ER^a^	PLN 97
	€ 23
Visits to hospital outpatient clinic^b^	PLN 18
	€ 4
Hospitalization of varicella case without complications^a^	PLN 2080
	€ 497
Hospitalization of varicella case with complications^a^	PLN 4004
	€ 956

*PLN* Polish złoty, *€* Euros

^a^Cost per case using this resource; based on the payments of the National Health Fund to the Regional Hospital Centre for Mother and Child in Poznań, Poland

^b^Cost per visit; based on an average salary for a specialized physician in the public system and an average of 25 patients/day

**Table 4:** Cost (PLN / €) per pediatric case of varicella

	Outpatients (*N* = 75)		Inpatients (*N* = 75)	
	Mean Cost^a^	95% CI	Mean Cost^a^	95% CI
Direct Costs				
Visits to doctor’s office	PLN 200.2	(186.5, 213.9)	PLN 124.8	(99.8, 149.7)
	€ 47.8	(44.6, 51.1)	€ 29.8	(23.9, 35.8)
Visits to ER	PLN 12.9	(4.5, 21.4)	PLN 5.2	(0.1, 10.2)
	€ 3.1	(1.1, 5.1)	€ 1.2	(0.0, 2.4)
Visits to hospital outpatient clinic	PLN 3.1	(1.5, 4.7)	PLN	
	€ 0.7	(0.4, 1.1)	€ 0.0	N/A
Hospitalization	PLN		PLN 3671.0	(N/C)
	€ N/A	N/A	€ 877.3	
ICU stay	PLN		PLN	N/A
	€ N/A	N/A	€ 0.0	
Prescription medications	PLN 10.1	(2.0, 18.2)	PLN 60.1	(43.4, 76.7)
	€ 2.4	(0.5, 4.3)	€ 14.4	(10.4, 18.3)
OTC medications	PLN 2.5	(2.0, 2.9)	PLN 2.0	(1.5, 2.5)
	€ 0.6	(0.5, 0.7)	€ 0.5	(0.4, 0.6)
Tests/procedures	PLN		PLN 113.2	(82.0, 144.3)
	€ 0.0	(N/C)	€ 27.0	(19.6, 34.5)
Allied health professional consultations	PLN		PLN 37.3	(21.4, 53.3)
	€ 0.0	(N/C)	€ 8.9	(5.1, 12.7)
Overall direct costs	PLN 228.8	(214.1, 243.4)	PLN 4013.5	(3953.1, 4073.9)
	€ 54.7	(51.2, 58.2)	€ 959.2	(944.8, 973.6)
Indirect Costs				
Lost work by caregivers	PLN 798.5	(630.4, 966.5)	PLN 999.8	(849.3, 1150.2)
	€ 190.8	(150.7, 231.0)	€ 238.9	(203.0, 274.9)
Total	PLN 1027.2	(859.5, 1195.0)	PLN 5013.3	(4820.0, 5206.5)
	€ 245.5	(205.3, 285.4)	€ 1198.1	(1151.3, 1243.6)

*PLN* Polish złoty, *€* Euros, *N/A* not applicable, *N/C* not calculable

^a^ Mean (95% CI) among all patients. Based on patients with available information

Table 5 presents the estimated annual costs (direct, indirect and total) associated with varicella among children < 15 years of age in Poland. These estimates were based on the cost per varicella case reported in Table 4, the number of varicella cases reported for 2015 in Poland (*n* = 187,518) [10] and the proportion of cases attributed to patients < 15 years of age (91.8%) reported for 2014 [21], the hospitalization rate in children with varicella for 2015 in Poland (0.71%), along with the proportion that were hospitalized in this age group (0.20%) using European hospitalization data reported for 2010 [33] and cases reported by age group for Poland [21]. Based on an estimated annual incidence of 172,117 pediatric (< 15 years of age) varicella cases, consisting of 350 inpatients and 171,768 outpatients, the total estimated annual direct and indirect costs associated with varicella in pediatric patients in Poland for 2015 are 40,699,037.9 PLN (€ 9,726,838.6) and 137,499,282.2 PLN (€ 32,861,546.3), respectively, for a total cost of 178,198,320.1 PLN (€ 42,588,384.9).

**Table 5:** Estimated annual (2015) costs (PLN / €) for children with varicella in Poland^a^

	Annual Cost (PLN / €)	(%) of Total Direct Cost
Direct Costs		
Visits to doctor’s office	PLN 34,429,801.2	85.4%
	€ 8,228,526.6	
Visits to ER	PLN 2,222,763.9	5.5%
	€ 531,227.9	
Visits to outpatient clinic	PLN 535,915.1	1.3%
	€ 128,080.6	
Hospitalization	PLN 1,283,974.3	2.3%
	€ 306,862.6	
ICU stay	PLN	0.0%
	€ 0.0	
Prescription medications	PLN 1,750,710.4	4.3%
	€ 418,409.8	
OTC medications	PLN 423,237.4	1.0%
	€ 101,151.3	
Tests/procedures	PLN 39,579.0	0.1%
	€ 9459.2	
Allied health professional consultations	PLN 13,056.6	0.0%
	€ 3120.5	
Total direct costs	PLN 40,699,037.9	N/A
	€ 9,726,838.6	
Indirect Costs		
Lost work by caregivers	PLN 137,499,282.2	N/A
	€ 32,861,546.3	
Total	PLN 178,198,320.1	
	€ 42,588,384.9	

*PLN* Polish złoty, *€* Euros, *N/A* not applicable

^a^Annual number of cases (n = 172,117) are estimated pediatric cases (< 15 years old) for 2015 based on the reports of the Polish National Institute of Public Health, and of the Department of Prevention and Control of Infectious Diseases, for the number of Polish varicella cases in 2014/2015 [10, 11]. The proportion of pediatric inpatients vs. outpatients was derived from the Polish hospitalization rate due to varicella [11], along with distribution of cases in each age group in Poland, estimated from the 2010 report of the European surveillance system for infectious diseases [33]

## Discussion

The findings of this study demonstrate that varicella infection among pediatric outpatients and inpatients in Poland is associated with considerable clinical and healthcare burden, as shown by the significant proportion of patients reporting complications and healthcare resource utilization, as well as the corresponding direct, indirect, and total costs.

With respect to clinical burden, 14.6% of outpatients vs. 82.6% of inpatients in our study experienced one or more complication. These results are consistent with previously published data for pediatric patients, which supports the external validity of our findings. Italy, Germany, and Switzerland reported complication rates of 3.5%, 5.9% and 12.0%, respectively, among patients presenting with varicella in an outpatient setting [35–37]. Amongst inpatients in Germany, Turkey, and Belgium, varicella-related complications were reported in 65.0%, 79.0%, and 79.6% of patients, respectively [38–40]. The most common types of complications we report for Poland included skin and soft tissue infection, dehydration, pneumonia, and cerebellitis, which are consistent with the commonly reported complications throughout European countries [41–49].

The results presented demonstrate a high degree healthcare resource use, with 92.0% of outpatients and 57.3% of inpatients visiting a doctor’s office at least once when they had varicella; among these patients, the average number of visits reported per outpatients and inpatients was 1.3 and 1.2 respectively. For inpatients, a mean hospital stay of 4.7 days was observed, which is supported by previous European data that reports three to 8 days [30, 39, 50–53]. These results also align with a recent systematic review which found that for Europe, in the absence of universal varicella vaccination, healthcare-related burden of varicella is substantial, reaching approximately 3 million pediatric cases per year, with overall annual number of varicella-related primary care physician consults and hospitalizations estimated at 3–3.9 million and 18,200–23,500 [33].

Glogowski et al. [54] previously estimated the cost of varicella for patients of all ages using data from 2000/2001, where they reported a total annual cost of 46,696,546 PLN. In this study, we report a total estimated annual cost of 178,198,320.1 PLN, which is almost four-fold the cost reported previously for Poland. Glogowski et al. based their calculations of direct cost on expert opinion, which included the assumption that each case of varicella used the following resources: 2 visits to the doctor’s office, use of antihistamines, analgesics, antipyretics, for all patients, 20% of patients receiving an antiviral, and 20% receiving an antibiotic. Whereas Glogowski et al. considered visits to the doctor’s office, medication, and hospitalizations as the only direct cost estimates, in this study we took into account additional expenses such as visits to the ER or outpatient clinics, tests/procedures, and allied health professional consultations. Our annual direct cost estimates totalled 40,699,037.9 PLN compared with 24,524,226.4 PLN in the earlier study, likely explained by the additional expenses included in our study. For indirect costs, Glogowski et al. assumed that only half of all varicella cases have one care-giver that takes time off work and of those taking time off, a total of 4 work days are missed. In our study, we assumed that for inpatients, the number of work days missed for each varicella case was equivalent to the average time spent in the hospital, which was 4.7 days. For outpatients, the number of work days missed was estimated as the duration of sick leave (time from 1st doctor’s note with indication to stay home to final one authorizing return to school), averaging out to approximately 2.7 days missed per outpatient case. The assumptions for yearly salary also differed between our study (46,203 PLN) and Glogowski et al. (24,742 PLN), which would be expected when comparing salaries over the span of 14 years. These estimates corresponded to a total annual estimated indirect cost of 137,499,282.2 PLN vs. 22,172,319.99 PLN reported previously for Poland. Even though it is possible that the indirect costs may have been overestimated in our study, this difference could be, in part, explained by increases in the cost of living, along with inflation.

Our cost estimates, although slightly higher, are in line with what is reported for other European countries. In Italy, the cost of varicella among children aged 1-14 years (uncomplicated cases) was estimated at $146.9 per child (equivalent to 481.6 PLN; cost in 1997 PLN [55]), while the average cost in our study was estimated at 1027.2 PLN (€ 245.5) per child for outpatient cases [35]. In Hungary, the average cost per child was reported to be 49,790.6 HUF (equivalent to 668.6 PLN; cost in 2015 PLN) for outpatient cases [56]. In Spain and Germany, estimated total cost per child (≤ 14 years and ≤ 12 years of age, respectively) was reported as € 108.67 (equivalent to 491.9 PLN; cost in 2004 PLN) [57] and € 162.5 (equivalent to 625.8 PLN; cost in 2002 PLN) [58], respectively, as compared to the estimated total cost of 1035.3 PLN (€ 247.7) in our study. The slightly higher cost observed in our study may be a reflection of differences in indirect costs, where Germany and Spain report estimates of € 105 (404.4 PLN in 2002) and € 74.9 (339.0 PLN in 2004) per case, respectively vs our estimate of 798.9 PLN (€ 190). Spain and Germany assumed that 0.97 and 0.7 work days were missed, respectively, for each case of varicella. However, in our study, we assumed that 4.7 days were missed for inpatients, and 2.7 days for outpatients, which may explain why our indirect costs are slightly higher in comparison.

A limitation of this study, inherent to the retrospective chart review design, is that only a cross-section of care sought may have been accounted for, thus resulting in the potential underestimation of the varicella associated HCRU and resultant costs. The lack of immunocompromised patients identified also represents a risk with respect to undervaluing the true per patient cost of varicella. The selection of only cases seeking medical consultation is an additional limitation, as it may have overestimated the burden associated with varicella. Even though the cost of healthcare resources is generally stable across Poland, minor variation might be expected between smaller and larger cities, particularly in the cost of visiting a doctor which, in turn, would result in minor variation in the estimated national cost. Finally, the relatively small sample size of the study, along with the small number of participating sites and the resulting limited geographical coverage, may have introduced selection bias and have reduced the generalizability of our analysis.

## Conclusions

Although generally considered a benign disease, substantial burden is associated with varicella in Poland, leading to increased resource utilization and considerable costs. When comparing the burden of varicella in Poland to other European countries such as Germany, Spain, and Italy, it is important to note that while the cost associated with varicella is generally similar, Poland is the only country among them to not include varicella vaccine in the national immunization program. As such, in providing local Polish data, the results of this study offer valuable perspectives into the real-world impact of pediatric varicella that may be used to foster discussions related to the potential benefits of universal VZV vaccination in Poland.

## Abbreviations

€: Euro
ACV: Acyclovir
CI: Confidence interval
EC: Ethics committee
ER: Emergency department
GPP: Good pharmacoepidemiology practice
HCRU: Healthcare resource utilization
HIV: Human immunodeficiency virus
ICU: Intensive care unit
MedDRA: Medical dictionary for regulatory activities
OECD: Organization for Economic Co-Operation
OTC: Over-the-counter
PLN: Polish złoty
SD: Standard deviation
VZV: Varicella-zoster virus
